# Analyses of Weight/Blood Pressure Changes before and after Tonsillectomy in Adults: A Longitudinal Follow-Up Study

**DOI:** 10.3390/ijerph18041948

**Published:** 2021-02-17

**Authors:** Jee Hye Wee, Chanyang Min, Dae Myoung Yoo, Min Woo Park, Chang Myeon Song, Bumjung Park, Hyo Geun Choi

**Affiliations:** 1 Department of Otorhinolaryngology-Head & Neck Surgery, Hallym University Sacred Heart Hospital, College of Medicine, Hallym University, Anyang 14068, Korea; weejh07@gmail.com (J.H.W.); bumjung426@gmail.com (B.P.); 2 Hallym Data Science Laboratory, College of Medicine, Hallym University, Anyang 14068, Korea; joicemin@naver.com (C.M.); ydm1285@naver.com (D.M.Y.); 3 Graduate School of Public Health, Seoul National University, Seoul 08826, Korea; 4 Department of Otorhinolaryngology-Head & Neck Surgery, Kangdong Sacred Heart Hospital, Seoul 05355, Korea; subintern@naver.com; 5 Department of Otorhinolaryngology-Head & Neck Surgery, College of Medicine, Hanyang University, Seoul 04763, Korea; cmsong@hanyang.ac.kr

**Keywords:** tonsillectomy, body weight changes, blood pressure, population surveillance

## Abstract

This study aimed to evaluate the changes in weight and blood pressure in Korean adults who underwent tonsillectomy compared to controls. A nested case-control study used data from the Korean National Health Insurance Service-Health Screening Cohort (2002–2015). Tonsillectomy was defined using claim code Q2300. The changes in weight and systolic/diastolic blood pressure (SBP/DBP) were measured before tonsillectomy and 1 year after tonsillectomy (study I) in some participants and during the second year after tonsillectomy (study II) in other participants. Patients who underwent tonsillectomy (*n* = 569 in study I; *n* = 556 in study II) were 1:4 matched with control participants (*n* = 2276 in study I; *n* = 2224 in study II). The paired *t*-test and linear mixed model were used to test the differences between groups at each time point. There were no changes in body mass index (*p* = 0.732 in study I; *p* = 0.128 in study II), SBP (*p* = 0.344 in study I; *p* = 0.559 in study II), or DBP (*p* = 0.826 in study I; *p* = 0.524 in study II) between the tonsillectomy patients and controls in the first or second year postoperatively. Subgroup analyses by age, sex, and degree of obesity showed consistent results. Tonsillectomy does not lead to a change in weight or blood pressure in Korean adults.

## 1. Introduction

Tonsillectomy is usually performed to treat infection (e.g., recurrent tonsillitis, peritonsillar abscess, tonsillolithiasis) and obstructive sleep apnea (OSA). A cross-sectional study reported that the epidemiologic trend in the reason for tonsillectomy shifted from infection to upper airway obstruction from 1970 to 2005 [1].

Several studies have reported that tonsillectomy and adenoidectomy (T&A) can be associated with weight gain and obesity in children. A systematic review showed that normal-weight and overweight children gained more weight than expected after T&A [2]. In a case-controlled study, children who received T&A, regardless of the indication for surgery, gained more weight than controls, especially in the obese group, and significantly increased their body mass index (BMI) z-scores compared with the control group at 2 years [3]. On the other hand, a recent study reported that children with obesity or overweight prior to T&A/tonsillectomy experienced a decrease in BMI z-score, whereas children who were underweight or normal weight experienced an increase in BMI z-score [4]. That study suggested that there are differences in BMI trajectory among patients in different BMI percentile categories after T&A/tonsillectomy.

Contrary to children’s studies, few studies have reported weight changes in adults after tonsillectomy. A previous retrospective study in the US reported that tonsillectomy led to weight loss between 12 and 40 days postoperatively, and patients older than 40 years (5.72 pounds/2.56 kg) lost the most weight but returned to baseline weight after approximately 5 months [5]. However, that study was limited in that the sample size was small and there was a lack of control groups, and it only showed short-term follow-up results.

In addition, several previous studies have addressed blood pressure changes after T&A in children with OSA, although conflicting results have been reported. In a Taiwanese cohort, nonobese children with OSA showed a reduced nocturnal diastolic blood pressure (DBP) index (mean difference, −3.3 mmHg; 95% confidence interval (CI) of difference, −5.6 to −1.1 mmHg) after T&A [6]. A randomized controlled trial of childhood OSA showed no significant changes in blood pressure parameters between the early T&A group and the watchful waiting group [7]. By contrast, when we reviewed PubMed and Embase using the terms “tonsillectomy and blood pressure” and restricted the search to only articles conducted in humans, the results showed that no studies have been conducted in adults. However, a previous study reported a transient increase in blood pressure after upper airway surgery for OSA [8]. A prospective case series showed that the average systolic blood pressure (SBP) and DBP decreased from 146 ± 15.3 mmHg to 122 ± 12.5 mmHg (*p* < 0.001) and from 91 ± 10.2 mmHg to 76 ± 7.8 mmHg (*p* < 0.001), respectively, in OSA patients after upper airway surgery [9]. However, those studies were also limited in that the sample size was small, there were no controls and included only OSA patients who underwent various surgical procedures.

The aim of this study was to evaluate the changes in BMI and SBP/DBP in patients who underwent tonsillectomy compared to controls in Korean adults. We also tried to further identify patient factors that influence changes in body weight and blood pressure.

## 2. Materials and Methods

### 2.1. Study Population

This study was approved by the Institutional Review Board of Hallym University (2019-01-003). The requirement for informed consent was waived considering the retrospective study design. The data of the Korean National Health Insurance Service-Health Screening Cohort were described in detail in a previous study [10].

The tonsillectomy participants were selected from among 514,866 individuals with 615,488,428 medical claim codes for procedures performed from 2002 through 2015 (*n* = 1321), and tonsillectomy participants who did not have both first-year follow-up and second-year follow-up data after tonsillectomy (*n* = 330) were excluded. Participants who did not undergo tonsillectomy from 2002 through 2015 (*n* = 513,545) were included in the control group. Because this health check-up was not performed annually in all participants, some tonsillectomy participants underwent a follow-up after the first year (*n* = 653, tonsillectomy I), and other tonsillectomy participants underwent a follow-up two years after tonsillectomy (*n* = 638, tonsillectomy II). Among all the participants, 300 tonsillectomy participants had both first- and second-year follow-up data. Therefore, we designed two studies.

**Figure 1:**
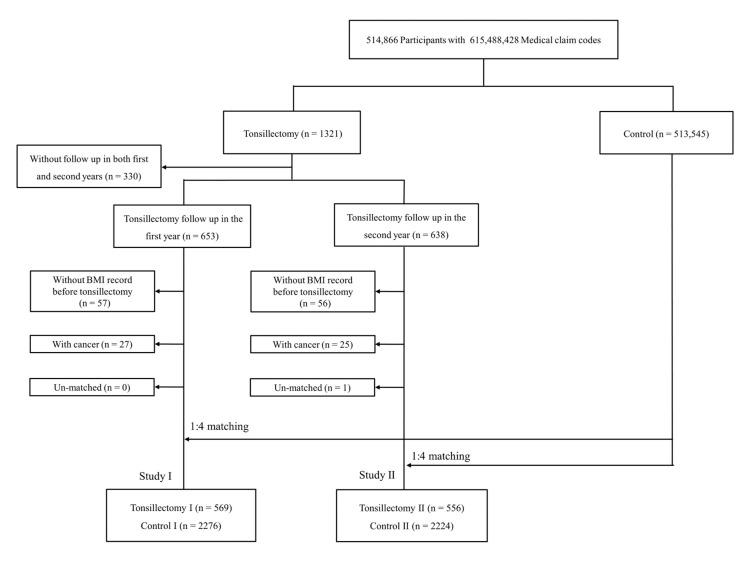
A flow chart of the participant selection process. Study I: among a total of 514,866 participants, 569 tonsillectomy participants were 1:4 matched with 2276 controls. Study II: among a total of 514,866 participants, 556 tonsillectomy participants were 1:4 matched with 2224 controls.

In studies I and II, tonsillectomy participants who did not have BMI data recorded before tonsillectomy (*n* = 57; *n* = 56) were excluded. Tonsillectomy participants who were diagnosed with cancer and underwent tonsillectomy were excluded (*n* = 27; *n* = 25). The tonsillectomy patients were matched 1:4 with the controls for age, sex, income, region of residence, and degree of obesity. The control participants were randomly selected to reduce selection bias. We set the index date of each tonsillectomy participant and their matched controls to the date on which tonsillectomy was performed. In the process of matching, 511,269/511,321 control participants were excluded. Finally, 569 tonsillectomy I and 556 tonsillectomy II participants were matched 1:4 with 2276 control I and 2224 control II participants (Figure 1).

### 2.2. Definition of Variables

Tonsillectomy was recorded if the participants were treated by procedures with claim codes Q2300. Weight/blood pressure (SBP/DBP) change after 1 year was defined as the change between the most recent BMI/blood pressure recorded before tonsillectomy and that recorded in the first year after tonsillectomy (study I). In addition, weight/blood pressure change after 2 years was defined as the change between the most recent BMI/blood pressure recorded before tonsillectomy and that recorded in the second year after tonsillectomy (study II).

### 2.3. Covariates

Age groups were created according to 5-year intervals: 40 to 44, 45 to 49, …, and 85 years old or older. Ten age groups were assigned. A total of 5 income groups were identified: (lowest–highest). The region of residence was considered either urban or rural.

Tobacco smoking was categorized based on the participant’s current smoking status (nonsmoker, past smoker, and current smoker). Alcohol consumption was categorized on the basis of the frequency of alcohol consumption (<1 time a week and ≥1 time a week). Degree of obesity was assessed using BMI (kg/m^2^). BMI values were grouped as follows based on the Asia-Pacific criteria proposed by the Western Pacific Regional Office (WPRO) in 2000: <18.5 (underweight), ≥18.5 to <23 (normal), ≥23 to <25 (overweight), ≥25 to <30 (obese I), and ≥30 (obese II) [11]. Fasting blood glucose and total cholesterol levels were obtained. Missing fasting blood glucose values (2/2845 (0.070%) in study I, 1/2780 (0.036%) in study II) and missing total cholesterol values (3/2845 (0.105%) in study I, 2/2780 (0.072%) in study II) were substituted with the average values of the variable among the finally selected participants.

The Charlson comorbidity index (CCI) is a widely used indicator to measure disease burden with 17 comorbidities. Cancer and metastatic cancer were excluded from the CCI score. The CCI was measured as a continuous variable from 0 (no) to 29 (multiple comorbidities) [12,13].

### 2.4. Statistics

The general characteristics between the tonsillectomy and control groups were compared. The chi-square test was used to compare the categorical variables, and the independent *t*-test was used to compare the continuous variables.

The differences between pre- and post-tonsillectomy were compared using paired *t*-tests. To analyze the interaction and estimated value, a linear mixed model was used. Age, sex, income, region of residence, tonsillectomy, and time of measurement were included as the independent variables and fixed effects. BMI, SBP/DBP, fasting blood glucose level, total cholesterol level, smoking status, alcohol consumption status, and CCI score were included as random effects. A first-order autoregressive model was selected as the repeated covariance type, considering the correlation of each participant’s iteration. The statistical analysis model of the linear mixed model that was used is as follows.

$$Y_{i}=X_{i1}\beta_{1\;}+\ldots +\;X_{ip}\beta_{p}+Z_{i1}u_{i}+\ldots +Z_{iq}u_{q}+e_{i},\;\mathrm{for}\;\mathrm{all}\;i=1,\ldots ,n\;$$

where Y = ($Y_{1},\ldots ,\;Y_{n}$)′, X corresponds to the n×p matrix of covariates with fixed effects $\beta \;=\;{\left(\beta_{1},\ldots ,\;\beta_{p}\right)}^{\prime }$, Z corresponds to the n×q matrix of covariates with random effects $u\;=\;{\left(u_{1},\ldots ,\;u_{q}\right)}^{\prime }\sim \;N\left(0,\tau I_{q}\right)$, and the residual error vector $e\;=\;{\left(e_{1},\ldots ,\;e_{n}\right)}^{\prime }\sim \;N{\left(0,\tau I_{n}\right)}^{\prime }$.

Subgroup analyses were performed by dividing the participants into age (<50 and ≥50 years old), sex (male and female) and degree of obesity (underweight, normal, overweight, obese I, and obese II).

Two-tailed analyses were conducted, and *p* values less than 0.05 were defined as statistically significant. SAS (v 9.4, SAS Institute Inc., Cary, NC, USA) was used for the statistical analyses.

## 3. Results

Table 1 shows the general characteristics of the participants in studies I and II. Age, sex, income, region of residence, and degree of obesity did not differ between matched participants (all *p* = 1.000). There was no difference in smoking status, alcohol consumption, fasting blood glucose level, total cholesterol level, or SBP/DBP between the tonsillectomy and control groups (all *p* > 0.05), except in the CCI score (*p* = 0.047) in study I and in smoking status (*p* = 0.041) and the CCI score (*p* = 0.004) in study II.

Table 2 shows the changes in the mean values of BMI and SBP/DBP from before to 1 year after tonsillectomy. The BMI did not change (25.14 ± 3.03 kg/m^2^ vs. 25.08 ± 2.98 kg/m^2^, *p* = 0.198) in the tonsillectomy group, while it decreased (25.05 ± 3.05 kg/m^2^ vs. 24.99 ± 2.99 kg/m^2^, *p* = 0.047) in the control group. However, the interaction effect of tonsillectomy×time on BMI did not reach statistical significance (*p* = 0.732). SBP (125.38 ± 13.95 mmHg vs. 123.85 ± 13.87 mmHg, *p* = 0.017) and DBP (79.68 ± 10.54 mmHg vs. 78.50 ± 10.19 mmHg, *p* = 0.018) decreased significantly in the tonsillectomy group, while SBP (125.45 ± 15.37 mmHg vs. 125.30 ± 15.08 mmHg, *p* = 0.647) and DBP (79.02 ± 10.53 mmHg vs. 78.65 ± 10.16 mmHg, *p* = 0.096) did not change in the control group. The interaction effect of tonsillectomy×time on SBP/DBP did not reach statistical significance (*p* = 0.344 for SBP, *p* = 0.826 for DBP). The subgroup analyses by age, sex, and degree of obesity (Table S1) also showed no difference between groups (all *p* > 0.05).

The changes in the mean values of BMI and SBP/DBP from before to 2 years after tonsillectomy are presented in Table 3. There were no changes in the mean BMI from before to after tonsillectomy in either group (*p* = 0.238; *p* = 0.378). There was a significant decrease in SBP (*p* = 0.012)/DBP (*p* = 0.001) in the tonsillectomy group and in DBP (*p* = 0.005) in the control group. However, the interaction effect of tonsillectomy×time on BMI and SBP/DBP did not reach statistical significance (*p* = 0.128 for BMI, *p* = 0.559 for SBP, *p* = 0.524 for DBP). In the subgroup analyses performed according to age and sex, a statistically significant difference was not found between the two groups in any of the analyses (all *p* > 0.05). In the subgroup analyses by the degree of obesity (Table S2), only the overweight group showed a significant difference in the BMI change between the two groups (*p* = 0.013), and no statistically significant difference was found between the two groups in any other analysis (*p* > 0.05).

## 4. Discussion

In this study, no significant difference was found in the changes in BMI or SBP/DBP between the tonsillectomy and control groups. In the subgroup analyses, the differences between the two groups were not statistically evident.

Tonsillectomy is a common surgery, and pain after tonsillectomy is one of the most noted problems and is often poorly controlled [14]. Oral pain can cause reduced oral intake, dysphagia, dehydration, and weight loss [15]. However, a previous study showed that tonsillectomy in adults leads to weight loss in the immediate postoperative period but returns to baseline weight within 6 months [5]. Consistent with these results, in the present study, there were no changes in BMI within the first year or second year after tonsillectomy.

While obesity is a leading cause of OSA [16], evidence suggests that OSA may cause weight gain [17], leading to the hypothesis that OSA treatment may help weight loss. However, in a meta-analysis of randomized trials, OSA treatment with continuous positive airway pressure (CPAP) led to a significant increase in BMI and body weight [18]. The authors explained that BMI and weight gain with OSA treatment are due to the complex determinants of weight. A normal body weight is known to be determined by a balance in catabolic and anabolic hormones, caloric intake, energy consumption, and physical activity. In addition, OSA treatment with CPAP has been reported to result in lower sympathetic activity [19] and leptin levels [20]. Because leptin induces the inhibition of appetite and increases basal energy expenditure, decreased leptin levels after OSA treatment can help explain the propensity for weight gain. Furthermore, increases in leptin levels in OSA patients have been linked to elevations in blood pressure [21]. In a meta-analysis, the mean net decreases in SBP (2.6 ± 0.6 mmHg) and DBP (2.0 ± 0.4 mmHg) were significant in the treatment with CPAP compared with passive treatment (*p* < 0.001) [22]. However, in the present study, even when OSA was treated with tonsillectomy, it did not affect weight change or blood pressure.

There are several limitations. First, we included patients who underwent tonsillectomy, but it was not known whether these participants had OSA or what the indications for surgery were. However, a meta-analysis in 2016 identified 17 studies with pre- and postoperative apnea–hypopnea index data in adults and concluded that isolated tonsillectomy is a successful treatment for OSA [23]. We divided the participants according to their degree of obesity. It is known that OSA and obesity have a bidirectional relationship, leading to a vicious cycle, where each factor results in the worsening of the other [24]. Nevertheless, in obese patients who are more likely to have OSA, their BMI decreased regardless of whether they underwent tonsillectomy. In addition, SBP decreased only in the tonsillectomy group, but there was no significant difference between the tonsillectomy and control groups. Second, the number of patients who underwent tonsillectomy was relatively small. Therefore, this study could have had inadequate statistical power. Third, hypertension was defined as SBP 140 mmHg or higher or DBP 90 mmHg or higher, but the mean blood pressure was not high in all participants (SBP = 125 mmHg; DBP = 79 mmHg). In a single study conducted in a Spanish population of normotensive subjects, the impact of CPAP did not result in a statistically significant reduction in the incidence of hypertension [25]. Additional studies are needed to compare the changes in blood pressure between hypertensive and nonhypertensive groups of patients who have been confirmed to have OSA by polysomnography.

Despite the limitations of this study, the results are important for a variety of reasons. First, a few previous studies have reported weight and blood pressure changes following tonsillectomy, but those studies lacked a control group and included relatively short follow-up periods. The present study included a control group and extended the follow-up duration to 2 years. These factors helped us determine whether changes in weight and blood pressure in adults were due to aging or tonsillectomy. Second, the results can be used as evidence for otolaryngologists to inform patients who undergo tonsillectomy of the postoperative results regarding weight and blood pressure changes. Third, the results may be used to educate patients on the benefits of lifestyle modifications, including a low-calorie diet. Some patients may expect to undergo weight loss after tonsillectomy, and we believe these results will help improve the treatment of OSA. However, there were no changes in BMI or SBP/DBP.

## 5. Conclusions

Tonsillectomy does not lead to a decrease in weight or blood pressure in Korean adults. These results can be used as evidence for otolaryngologists to inform patients who undergo tonsillectomy of the postoperative results regarding weight and blood pressure changes.

## Figures and Tables

**Table 1:** General characteristics of participants.

Characteristics	Study I			Study II		
	Tonsillectomy I	Control I	*p*-Value	Tonsillectomy II	Control II	*p*-Value
Age (years old, *n*, %)			1			1
40–44	56 (9.8)	224 (9.8)		61 (11.0)	244 (11.0)	
45–49	167 (29.4)	668 (29.4)		161 (29.0)	644 (29.0)	
50–54	167 (29.4)	668 (29.4)		189 (34.0)	756 (34.0)	
55–59	126 (22.1)	504 (22.1)		95 (17.1)	380 (17.1)	
60–64	38 (6.7)	152 (6.7)		36 (6.5)	144 (6.5)	
65–69	13 (2.3)	52 (2.3)		11 (2.0)	44 (2.0)	
70–74	1 (0.2)	4 (0.2)		2 (0.4)	8 (0.4)	
75–79	1 (0.2)	4 (0.2)		1 (0.2)	4 (0.2)	
Sex (*n*, %)			1			1
Male	413 (72.6)	1652 (72.6)		409 (73.6)	1636 (73.6)	
Female	156 (27.4)	624 (27.4)		147 (26.4)	588 (26.4)	
Income (*n*, %)			1			1
1 (lowest)	47 (8.3)	188 (8.3)		47 (8.5)	188 (8.5)	
2	57 (10.0)	228 (10.0)		50 (9.0)	200 (9.0)	
3	57 (10.0)	228 (10.0)		61 (11.0)	244 (11.0)	
4	115 (20.2)	460 (20.2)		115 (20.7)	460 (20.7)	
5 (highest)	293 (51.5)	1172 (51.5)		283 (50.9)	1132 (50.9)	
Region of residence (*n*, %)			1			1
Urban	284 (49.9)	1136 (49.9)		275 (49.5)	1100 (49.5)	
Rural	285 (50.1)	1140 (50.1)		281 (50.5)	1124 (50.5)	
Obesity (*n*, %)			1			1
Underweight	3 (0.5)	12 (0.5)		2 (0.4)	8 (0.4)	
Normal	124 (21.8)	496 (21.8)		129 (23.2)	516 (23.2)	
Overweight	161 (28.3)	644 (28.3)		156 (28.1)	624 (28.1)	
Obese I	244 (42.9)	976 (42.9)		230 (41.4)	920 (41.4)	
Obese II	37 (6.5)	148 (6.5)		39 (7.0)	156 (7.0)	
Smoking status (*n*, %)			0.49			0.041 ^1^
Nonsmoker	323 (56.8)	1296 (56.9)		327 (58.8)	1322 (59.4)	
Past smoker	113 (19.9)	409 (18.0)		111 (20.0)	355 (16.0)	
Current smoker	133 (23.4)	571 (25.1)		118 (21.2)	547 (24.6)	
Alcohol consumption (*n*, %)			0.606			0.105
<1 time a week	344 (60.5)	1334 (58.6)		345 (62.1)	1296 (58.3)	
≥1 time a week	225 (39.5)	942 (41.4)		211 (38.0)	928 (41.7)	
Fasting blood glucose (*n*, %)			0.958			0.957
<100 mg/dL	361 (63.4)	1441 (63.3)		357 (64.2)	1440 (64.8)	
100–125 mg/dL	168 (29.5)	667 (29.3)		158 (28.4)	618 (27.8)	
≥126 mg/dL	40 (7.0)	168 (7.4)		41 (7.4)	166 (7.5)	
Total cholesterol (*n*, %)			0.282			0.372
<200 mg/dL	301 (52.9)	1186 (52.1)		293 (52.7)	1172 (52.7)	
200–239 mg/dL	205 (36.0)	782 (34.4)		179 (32.2)	762 (34.3)	
≥240 mg/dL	63 (11.1)	308 (13.5)		84 (15.1)	290 (13.0)	
CCI score (*n*, %)			0.047 ^1^			0.004 ^1^
0	444 (78.0)	1875 (82.4)		427 (76.8)	1859 (83.6)	
1	92 (16.2)	314 (13.8)		97 (17.5)	280 (12.6)	
2	24 (4.2)	53 (2.3)		24 (4.3)	57 (2.6)	
3	05 (0.9)	24 (1.1)		6 (1.1)	19 (0.9)	
≥4	4 (0.7)	10 (0.4)		2 (0.4)	9 (0.4)	
Systolic blood pressure (mean, SD) ^2^	125.38 ± 13.95	125.45 ± 15.37	0.93	125.49 ± 14.80	125.61 ± 15.84	0.869
Diastolic blood pressure (mean, SD) ^2^	79.68 ± 10.54	79.02 ± 10.53	0.181	79.41 ± 10.79	79.41 ± 10.71	0.995

BMI, body mass index; CCI, Charlson comorbidity index; SD, standard deviation; ^1^ Chi-square test. Significance at *p* < 0.05; ^2^ Independent *t*-test. Significance at *p* < 0.05.

**Table 2:** Difference in mean values of BMI and blood pressure between pre and 1-year post tonsillectomy in study I according to age and sex.

Characteristics	Tonsillectomy I			Control I			Interaction ^3^	Linear Mixed Model ^5^	
	Previous (Mean, SD)	Post 1 yr (Mean, SD)	*p*-Value ^1^	Previous (Mean, SD)	Post 1 yr (Mean, SD)	*p*-Value ^1^	*p*-Value	EV ^4^	*p*-Value ^2^
Total participants (*n* = 2845)									
BMI	25.14 ± 3.03	25.08 ± 2.98	0.198	25.05 ± 3.05	24.99 ± 2.99	0.047 ^1^	0.732	0.070	0.609
SBP	125.38 ± 13.95	123.85 ± 13.87	0.017 ^1^	125.45 ± 15.37	125.30 ± 15.08	0.647	0.344	−0.808	0.064
DBP	79.68 ± 10.54	78.50 ± 10.19	0.018 ^1^	79.02 ± 10.53	78.65 ± 10.16	0.096	0.826	0.687	0.022 ^2^
Age <50 years old, men (*n* = 905)									
BMI	25.08 ± 2.86	25.19 ± 2.81	0.224	24.91 ± 2.75	24.95 ± 2.74	0.331	0.252	0.147	0.513
SBP	126.45 ± 13.50	124.75 ± 13.62	0.103	125.27 ± 14.94	125.08 ± 14.44	0.736	0.734	−0.684	0.345
DBP	81.80 ± 10.19	80.20 ± 10.09	0.059	80.04 ± 10.76	79.63 ± 10.41	0.315	0.621	1.039	0.048 ^2^
Age < 50 years old, women (*n* = 210)									
BMI	24.08 ± 3.32	23.99 ± 3.41	0.715	24.06 ± 3.45	24.06 ± 3.44	0.944	0.715	−0.005	0.993
SBP	120.05 ± 11.93	120.38 ± 13.49	0.881	120.33 ± 16.16	118.80 ± 16.01	0.186	0.355	−1.069	0.510
DBP	75.50 ± 9.35	75.05 ± 8.59	0.756	74.80 ± 10.36	74.30 ± 10.62	0.539	0.536	0.752	0.497
Age ≥50 years old, men (*n* = 1160)									
BMI	25.55 ± 2.95	25.38 ± 2.86	0.035 ^1^	25.44 ± 3.12	25.30 ± 2.95	0.008 ^1^	0.952	0.092	0.668
SBP	126.55 ± 14.13	124.52 ± 13.26	0.061	127.23 ± 14.35	127.25 ± 14.65	0.965	0.066	−0.900	0.172
DBP	80.28 ± 10.82	79.44 ± 9.95	0.324	80.15 ± 9.88	79.74 ± 9.84	0.239	0.312	0.525	0.260
Age ≥50 years old, women (*n* = 570)									
BMI	24.80 ± 3.23	24.67 ± 3.21	0.302	24.83 ± 3.09	24.77 ± 3.16	0.377	0.881	−0.005	0.986
SBP	123.29 ± 14.43	122.32 ± 15.41	0.503	124.00 ± 17.13	124.09 ± 15.77	0.901	0.783	−0.633	0.564
DBP	76.61 ± 9.87	75.17 ± 10.39	0.160	76.65 ± 10.76	76.46 ± 9.44	0.698	0.374	0.292	0.672

BMI, body mass index; CCI, Charlson comorbidity index; EV, Estimated value; SBP, systolic blood pressure; DBP, diastolic blood pressure; ^1^ Paired *t*-test, significance at *p* < 0.05; ^2^ Linear mixed model, significance at *p* < 0.05; ^3^ Interaction effects between time and group. ^4^ Estimated value of linear mixed model for tonsillectomy I group based on the control I group. ^5^ Fixed effects were age, sex, income, region of residence, tonsillectomy, and time of measurement. Random effects were BMI, systolic blood pressure, diastolic blood pressure, fasting blood glucose, total cholesterol, smoking, alcohol consumption, and CCI scores.

**Table 3:** Difference in mean values of BMI and blood pressure between pre and 2-year post tonsillectomy in study II according to age and sex.

Characteristics	Tonsillectomy II			Control II			Interaction ^3^	Linear Mixed Model ^5^	
	Previous (Mean, SD)	Post 2 yr (Mean, SD)	*p*-Value ^1^	Previous (Mean, SD)	Post 2 yr (Mean, SD)	*p*-Value ^1^	*p*-Value	EV ^4^	*p*-Value ^2^
Total participants (*n* = 2780)									
BMI	25.14 ± 3.04	25.21 ± 3.06	0.238	25.05 ± 3.02	25.03 ± 3.07	0.378	0.128	0.039	0.783
SBP	125.49 ± 14.80	123.81 ± 13.95	0.012 ^1^	125.61 ± 15.84	125.32 ± 14.57	0.388	0.559	−0.184	0.681
DBP	79.41 ± 10.79	77.76 ± 10.08	0.001 ^1^	79.41 ± 10.71	78.72 ± 10.13	0.005 ^1^	0.524	−0.180	0.561
Age <50 years old, men (*n* = 890)									
BMI	25.42 ± 2.78	25.45 ± 2.68	0.778	25.41 ± 2.93	25.36 ± 3.00	0.338	0.506	−0.047	0.840
SBP	125.97 ± 13.62	124.81 ± 14.59	0.318	125.87 ± 15.27	125.19 ± 13.73	0.231	0.852	−0.297	0.685
DBP	80.97 ± 9.76	79.70 ± 10.75	0.144	80.62 ± 10.30	79.69 ± 9.82	0.024 ^1^	0.911	0.355	0.488
Age <50 years old, women (*n* = 220)									
BMI	23.72 ± 2.98	23.90 ± 2.91	0.410	23.88 ± 3.11	24.02 ± 3.16	0.103	0.802	−0.165	0.743
SBP	117.02 ± 14.18	118.41 ± 16.70	0.523	118.19 ± 15.51	119.72 ± 14.51	0.189	0.925	−1.217	0.410
DBP	74.00 ± 11.14	74.34 ± 9.82	0.827	73.84 ± 10.70	74.47 ± 9.30	0.441	0.831	0.828	0.411
Age ≥50 years old, men (*n* = 1155)									
BMI	25.26 ± 2.94	25.32 ± 2.95	0.518	25.12 ± 2.87	25.06 ± 2.92	0.112	0.275	0.116	0.582
SBP	126.99 ± 14.34	124.15 ± 13.30	0.008 ^1^	127.06 ± 14.99	126.81 ± 14.26	0.625	0.361	0.030	0.965
DBP	80.21 ± 10.74	77.77 ± 9.52	0.003 ^1^	80.29 ± 10.74	79.59 ± 9.95	0.072	0.516	−0.015	0.976
Age ≥50 years old, women (*n* = 515)									
BMI	24.98 ± 3.55	25.11 ± 3.80	0.445	24.78 ± 3.33	24.82 ± 3.37	0.558	0.557	0.065	0.860
SBP	124.92 ± 16.86	123.64 ± 12.61	0.418	125.09 ± 17.81	124.59 ± 16.06	0.581	0.929	0.181	0.874
DBP	77.26 ± 11.55	75.83 ± 9.57	0.237	77.74 ± 10.42	76.92 ± 10.69	0.161	0.819	−0.402	0.586

BMI, body mass index; CCI, Charlson comorbidity index; EV, Estimated value; SBP, systolic blood pressure; DBP, diastolic blood pressure; ^1^ Paired *t*-test, Significance at *p* < 0.05; ^2^ Linear mixed model, Significance at *p* < 0.05; ^3^ Interaction effects between time and group. ^4^ Estimated value of linear mixed model for tonsillectomy II group based on the control II group. ^5^ Fixed effects were age, sex, income, region of residence, tonsillectomy, and time of measurement. Random effects were BMI, systolic blood pressure, diastolic blood pressure, fasting blood glucose, total cholesterol, smoking, alcohol consumption, and CCI scores.

## Data Availability

The data presented in this study are available from the Korea National Health Insurance Sharing Service (https://nhiss.nhis.or.kr) subject to their requirements and fees.

## Supplementary Materials

The following are available online at https://www.mdpi.com/1660-4601/18/4/1948/s1, Table S1: Difference in mean values of BMI and blood pressure between pre and 1-year post of tonsillectomy in tonsillectomy I and control I group according to obesity, Table S2: Difference in mean values of BMI and blood pressure between pre and 2-year post of tonsillectomy in tonsillectomy II and control II group according to obesity.
