# Supplementary material for: Analyses of Weight/Blood Pressure Changes before and after Tonsillectomy in Adults: A Longitudinal Follow-Up Study
Source: Int J Environ Res Public Health. 2021 Feb 17;18(4):1948. doi: 10.3390/ijerph18041948 (PMC7921988; doi:10.3390/ijerph18041948)
Supplement: Supplementary file 1 [file ijerph-18-01948-s001.pdf]

Table S1. Difference in mean values of BMI and blood pressure between pre and 1-year post of tonsillectomy in tonsillectomy I and control I group according to obesity

Characteristics	Tonsillectomy I			Control I			Interaction†	Linear mixed model §	
	Previous (mean, SD)	Post 1yr (mean, SD)	P-value*	Previous (mean, SD)	Post 1yr (mean, SD)	P-value*	P-value	EV ‡	P-value**
Underweight (n = 15)									
BMI	18.04 ± 0.46	18.60 ± 1.39	0.472	17.82 ± 0.64	18.09 ± 1.07	0.347	0.646	0.218	0.709
SBP	110.00 ± 10.00	111.00 ± 16.52	0.858	111.33 ± 9.58	114.33 ± 13.10	0.433	0.710	-4.937	0.174
DBP	70.00 ± 10.00	70.33 ± 9.50	0.957	68.58 ± 7.27	68.58 ± 7.87	1.000	0.711	3.985	0.116
Normal (n = 620)									
BMI	21.30 ± 1.08	21.66 ± 1.53	<0.001*	21.40 ± 1.12	21.58 ± 1.43	<0.001*	0.086	-0.105	0.404
SBP	119.50 ± 13.69	120.12 ± 12.53	0.625	120.24 ± 15.30	120.16 ± 14.41	0.894	0.645	0.005	0.995
DBP	75.57 ± 9.77	76.02 ± 9.06	0.651	76.14 ± 10.45	75.43 ± 10.00	0.125	0.335	-0.241	0.706
Overweight (n = 805)									
BMI	24.06 ± 0.59	24.09 ± 1.37	0.711	23.98 ± 0.58	24.09 ± 1.16	0.009*	0.453	0.080	0.340
SBP	124.91 ± 13.16	123.74 ± 13.90	0.334	123.67 ± 14.10	123.61 ± 14.02	0.917	0.825	-0.522	0.508
DBP	79.60 ± 10.60	78.35 ± 9.87	0.175	77.88 ± 10.14	77.90 ± 9.54	0.974	0.342	0.980	0.078
Obese I (n = 1,220)									
BMI	26.91 ± 1.37	26.67 ± 1.70	0.002*	26.66 ± 1.21	26.47 ± 1.66	<0.001*	0.671	0.249	0.016**
SBP	127.43 ± 13.39	125.43 ± 14.42	0.049*	128.28 ± 15.05	128.01 ± 15.05	0.581	0.567	-1.705	0.012**
DBP	81.21 ± 10.33	79.73 ± 10.79	0.063	80.45 ± 10.27	80.13 ± 10.11	0.348	0.680	1.159	0.013**
Obese II (n = 185)									
BMI	31.62 ± 1.38	30.82 ± 2.32	0.007*	31.85 ± 3.24	31.13 ± 2.80	0.010*	0.957	-0.301	0.562
SBP	134.92 ± 13.46	127.35 ± 11.50	0.004*	133.03 ± 16.03	132.92 ± 14.99	0.932	0.087	2.532	0.148
DBP	84.46 ± 9.74	80.05 ± 9.60	0.028*	84.99 ± 10.17	83.72 ± 9.64	0.157	0.589	-1.595	0.167

BMI, body mass index; CCI, Charlson comorbidity index; EV, Estimated value; SBP, systolic blood pressure; DBP, diastolic blood pressure

* Paired t-test, Significance at $P < 0.05$

** Linear mixed model, Significance at $P < 0.05$

† Interaction effects between time and group.

‡ Estimated value of linear mixed model for tonsillectomy I group based on the control I group.

§ Fixed effects were age, sex, income, region of residence, tonsillectomy, and time of measurement. Random effects were BMI, systolic blood pressure, diastolic blood pressure, fasting blood glucose, total cholesterol, smoking, alcohol consumption, and CCI scores.

Table S2. Difference in mean values of BMI and blood pressure between pre and 2-year post of tonsillectomy in tonsillectomy II and control II group according to obesity

Characteristics	Tonsillectomy II			Control II			Interaction †	Linear mixed model §	
	Previous (mean, SD)	Post 2yr (mean, SD)	P-value*	Previous (mean, SD)	Post 2yr (mean, SD)	P-value*	P-value	EV ‡	P-value**
Underweight (n = 10)									
BMI	17.99 ± 0.68	19.05 ± 0.62	0.025*	17.78 ± 0.45	18.68 ± 1.14	0.025*	0.813	0.181	0.787
SBP	135.00 ± 21.21	120.00 ± 0.00	0.500	124.88 ± 23.00	121.13 ± 12.45	0.623	0.766	11.121	0.125
DBP	85.00 ± 7.07	75.50 ± 6.36	0.500	82.13 ± 21.42	77.38 ± 9.72	0.459	0.091	-6.539	0.168
Normal (n = 645)									
BMI	21.44 ± 1.09	21.76 ± 1.47	0.002*	21.46 ± 1.13	21.73 ± 1.54	<0.001*	0.604	-0.037	0.771
SBP	118.33 ± 14.24	118.61 ± 13.12	0.819	119.17 ± 14.66	120.85 ± 14.15	0.012*	0.996	-0.522	0.547
DBP	74.90 ± 10.06	74.01 ± 9.43	0.329	75.33 ± 10.17	75.81 ± 10.23	0.315	0.477	0.134	0.829
Overweight (n = 780)									
BMI	24.12 ± 0.58	24.43 ± 1.36	0.003*	24.00 ± 0.56	24.04 ± 1.27	0.433	0.013†	0.116	0.193
SBP	124.28 ± 14.56	123.39 ± 12.91	0.478	124.74 ± 15.03	124.82 ± 14.15	0.905	0.658	0.665	0.430
DBP	77.79 ± 10.70	76.81 ± 9.45	0.321	78.87 ± 9.95	78.29 ± 9.59	0.170	0.954	-0.849	0.139
Obese I (n = 1,150)									
BMI	26.84 ± 1.31	26.67 ± 1.75	0.069	26.70 ± 1.22	26.53 ± 1.70	<0.001*	0.968	0.136	0.214
SBP	128.88 ± 13.57	125.71 ± 14.37	0.003*	129.15 ± 16.04	127.42 ± 14.53	0.002*	0.945	0.710	0.111
DBP	82.26 ± 9.97	79.68 ± 10.15	0.001*	81.51 ± 10.81	80.19 ± 10.12	0.001*	0.432	0.915	0.061
Obese II (n = 195)									
BMI	31.82 ± 1.64	31.42 ± 2.54	0.174	31.82 ± 2.10	31.36 ± 2.76	0.010*	0.859	-0.009	0.983
SBP	134.10 ± 14.10	132.00 ± 12.30	0.506	129.59 ± 13.92	129.92 ± 14.20	0.807	0.312	3.218	0.063
DBP	83.90 ± 11.76	82.59 ± 9.91	0.615	82.58 ± 9.82	81.47 ± 9.64	0.256	0.508	-1.031	0.408

BMI, body mass index; CCI, Charlson comorbidity index; EV, Estimated value; SBP, systolic blood pressure; DBP, diastolic blood pressure

* Paired t-test, Significance at $P < 0.05$

** Linear mixed model, Significance at $P < 0.05$

† Interaction effects between time and group.

‡ Estimated value of linear mixed model for tonsillectomy II group based on the control II group.

§ Fixed effects were age, sex, income, region of residence, tonsillectomy, and time of measurement. Random effects were BMI, systolic blood pressure, diastolic blood pressure, fasting blood glucose, total cholesterol, smoking, alcohol consumption, and CCI scores.
